# COVID-19 and malaria, a tale of two diseases

**DOI:** 10.1016/j.ebiom.2021.103554

**Published:** 2021-08-24

**Authors:** EBioMedicine

Malaria has been with us since ancient times, with Hippocrates believing the disease was spread by corrupted air. It was centuries until the true cause of the disease was identified. On Aug 20, 1894, which is now recognised as World Mosquito Day, Sir Ronald Ross showed that female mosquitoes were the vector responsible for transmitting plasmodium parasites, the causative agent of malaria. This discovery was a crucial step in the continuing global efforts to eradicate the disease, efforts that might be stalled (or reversed) by a much more recent disease, COVID-19.

COVID-19 related disruption, lockdowns and travel restrictions prevent front-line health-care workers from reaching areas of need and break supply chains of antimalarial drugs. A modelling study published in *The Lancet Infectious Diseases* predicted that COVID-19 related disruption, which caused a 25% reduction in antimalarial drug coverage, could have led to as many as 487 900 deaths across Africa in 2020 (compared with 386 400 predicted deaths with a 0% reduction in antimalarial drug coverage). Cases of malaria would similarly rise.

With this disruption, the need for innovative techniques to diagnose, treat, and prevent malaria is more important than ever. One such innovation, published in *EBioMedicine*, is a proof-of-concept diagnostic technique to detect *Plasmodium falciparum*. Current detection methods use rapid diagnostic tests that detect plasmodium proteins in blood. However, such tests are unable to detect low density infections (ie, <200 parasites per μl of blood). This new method exploits a CRISPR-based technique named SHERLOCK (Specific High-Sensitivity Enzymatic Reporter UnLOCKing). First, plasmodium DNA is isolated from a patient and amplified using recombinase polymerase amplification (RPA, an isothermal cousin to PCR). The RPA product is then converted to single-stranded RNA (ssRNA) by in vitro transcription. Finally, the ssRNA is recognised by a Cas13 protein which, like Cas9, uses a guide RNA to bind target sequences (in this case, a region of the *P falciparum* genome converted to ssRNA). The activity of Cas13 RNase is activated upon binding the target sequence, resulting in the cleavage of fluorescent reporter RNA molecules that produce a light signal, confirming the presence of *P falciparum* DNA. SHERLOCK has been able to deduce the presence of plasmodium DNA in both clinical samples and in mosquitoes. Compared with real-time PCR methods, the SHERLOCK technique achieved 94% sensitivity and 94% specificity across 123 clinical samples. Modifying the guide RNA of Cas13 allows the assay to detect the single nucleotide variant (A581G) in the plasmodium dihydropteroate synthase gene, associated with sulfadoxine resistance.

However, malaria parasites do not only display sulfadoxine resistance. Two recent studies in *Nature Medicine* and *The Lancet Infectious Diseases*, provide evidence that artemisinin resistance (previously confined to southeast Asia) is present in Rwanda. Complete resistance would be devasting and new antimalarial drugs are urgently needed. A novel antimalarial, 2-aminobenzimidazole (2-AB), has been reported in *European Journal of Medicinal Chemistry*. The study reported the synthesis of a series of 2-ABs that showed cytotoxicity against *P falciparum* in vitro without damaging human cells. Most notably 2-ABs showed toxicity against strains of *P falciparum* resistant to chloroquine and artemisinin. The drugs also showed slow metabolism in a human liver model, suggesting low clearance in patients. Such findings require confirmation in vivo but these early results are encouraging.

One way to prevent drug resistance is to prevent the need for treatment by implementing vaccination programmes. Compared with the several available vaccines for COVID-19, only a single vaccine, RTS,S, is approved for malaria. RTS,S was approved by the European Medicines Agency and the efficacy of the vaccine in children was shown in 2015 in a phase 3 trial published in *The Lancet*. Pilot projects for the real-world efficacy of the vaccine were launched in Malawi, Ghana, and Kenya in 2019 (unpublished at the time of writing). As with COVID-19, there would be advantages to having multiple vaccines. In June, 2021, a potential new vaccine was published in *Nature*. Participants were inoculated with cryopreserved and still infectious *P falciparum* under prophylactic cover with either pyrimethamine (PYR) or chloroquine (CQ). PYR and CQ, respectively, kill the liver-stage and blood-stage of the parasite. Patients then underwent controlled infections with untreated *P falciparum*. The PYR vaccine prevented malaria in fourteen out of seventeen individuals and the CQ vaccine offered protection in all six participants tested. Larger trials will be required to determine the vaccine's effectiveness.

At the time of writing, WHO estimates that, since the emergence of COVID-19 in December, 2019, there have been 198 million recorded cases of COVID-19 worldwide; a truly staggering number, but still less than the 229 million new malaria cases estimated to have occurred in 2019 alone. Despite these two numbers, the funding situations for these diseases are widely disparate. Devex, a media platform devoted to international development, estimated that (as of June 27, 2020) approximately US$469 billion had been devoted globally towards just health systems, treatment and vaccines to tackle COVID-19, with the total amount to combat COVID-19 exceeding $21.7 trillion. In comparison, the Global Fund, an international financing organisation that provides 56% of all international malaria funding has, as of August, 2020, invested $13.5 billion to combat malaria since 2002. Why the difference in resources in combatting malaria and COVID-19? One possibility is that COVID-19 affects those with access to funds (ie, high-income countries), as opposed to malaria, which is largely confined to low-income countries.

This line of thinking might be short-sighted. A modelling study in *The Lancet Planetary Health* found that, because of climate change, the epidemic regions for malaria will creep towards temperate areas if the world continues to warm between now and 2070. However, COVID-19 has shown that with sufficient political willpower and resources, great strides in disease prevention and treatment can be made in a short space of time. Hopefully, in the future, some of the lessons learned during the COVID-19 pandemic can be used against malaria.

